# Supplementary material for: Testing the reliability and validity of a newly graduated nurses’ teaching experience scale
Source: PLoS One. 2026 Feb 20;21(2):e0343270. doi: 10.1371/journal.pone.0343270 (PMC12923036; doi:10.1371/journal.pone.0343270)
Supplement: S1 Table — (PDF) [file pone.0343270.s001.pdf]

Table 1. Summary of the participants (n=157)

Items		n (%)
Sex	Female	149 (94.9)
	Male	8 (5.1)
Age (average ± standard deviation)		25.4±3.3
Licenses	Nurse	157(100.0)
	Public health nurse	31(19.7)
	Midwife	1(0.6)
	Others	1(0.6)*
		* school nurse
Highest level of education in nursing (Number of years required for basic nursing education)	Upper secondary school major (5 years)	8(5.1)
	Vocational school (3 years)	84(53.5)
	Junior college (2 years)	5(3.2)
	University (or college) (4 years)	58(36.9)
	Graduate school	1(0.6)
	N/A	1(0.6)
Years of nursing experience	3rd year	74 (47.1)
	4th year	65 (41.4)
	5th year	18 (11.5)
