# Supplementary material for: Testing the reliability and validity of a newly graduated nurses’ teaching experience scale
Source: PLoS One. 2026 Feb 20;21(2):e0343270. doi: 10.1371/journal.pone.0343270 (PMC12923036; doi:10.1371/journal.pone.0343270)
Supplement: S2 Table — (PDF) [file pone.0343270.s002.pdf]

Table 2. Exploratory factor analysis of the Newly Graduated Nurses Teaching Experience Scale (n=157)

		Factor				
		1	2	3	4	5
Factor 1 Experience of receiving cooperation from others (Cronbach's α =.923)	1 Seniors directly communicated with me about the NGNs' growth.	**.905**	-.216	.077	-.125	.064
	2 Seniors sympathized with my experience teaching NGNs.	**.849**	.163	-.078	.027	-.161
	3 Seniors asked me if I had any problems while teaching NGNs.	**.795**	-.041	-.159	-.049	.049
	4 I received advice from seniors on how to instruct NGNs.	**.785**	.095	-.009	.014	-.060
	5 I talked to seniors about my problems with teaching.	**.763**	.102	-.090	.054	-.023
	6 I consulted with my seniors about specific guidance methods for NGNs.	**.754**	-.032	.143	.129	-.067
	7 I asked my seniors to provide guidance to NGNs when I was not available.	**.601**	-.098	.104	.036	.237
	8 Nurses with less experience in teaching newly graduated nurses encouraged each other.	**.477**	-.010	.099	-.045	.256
Factor 2 Experience of being there for the NGNs (Cronbach's α =.854)	9 I tried to create an atmosphere where NGNs felt comfortable talking to me about things other than work.	-.088	**.845**	-.088	-.192	-.061
	10 I tried to empathize with NGNs' stories.	-.021	**.699**	-.052	.017	-.196
	11 I told NGNs that I could be a consultant for them.	.030	**.648**	-.032	-.017	.189
	12 I paid attention to the relationship between the NGNs and other staffs.	.052	**.638**	-.015	.185	.010
	13 I ensured that the NGNs and I could openly express our thoughts to each other.	-.007	**.602**	.225	.007	.023
	14 I felt that I was in charge of the NGNs and that I cared about the NGNs.	.031	**.550**	.174	-.073	.147
	15 I made time for the NGNs to tell me what they were worried about in their work.	.080	**.487**	-.020	.009	.272
Factor 3 Experience of feeling positive about teaching (Cronbach's α =.865)	16 I felt that my perspective on education was broadened.	.079	-.037	**.896**	-.009	-.088
	17 I felt glad to have been involved in the training NGNs.	.001	.041	**.831**	-.123	-.026
	18 I gained confidence in education.	-.179	-.125	**.791**	-.015	.113
	19 Through the NGNs' guidance, I came to recognize myself as I am now.	-.029	.220	**.648**	.179	-.104
	20 I could see positive changes in NGNs through my guidance.	.101	.003	**.548**	-.041	.008
Factor 4 Experience of struggling with teaching (Cronbach's α =.849)	21 I was troubled because I could not understand NGNs' ideas.	-.001	-.071	-.030	**.870**	-.021
	22 I had a difficult time communicating with NGNs.	-.123	-.043	-.020	**.854**	.073
	23 I was troubled because I could not observe the NGNs' positive attitude toward learning.	.105	.080	-.110	**.735**	-.023
	24 I was troubled by the teaching method due to differences from the basic education course.	.069	-.040	.113	**.549**	.044
Factor 5 Experience of teaching according to level of achievement	25 Goals were set/modified each time based on the NGNs' achievement status.	.057	-.049	-.112	-.021	**1.036**
	26 Progress of knowledge and skills was checked with the NGNs.	.045	.083	.000	-.004	**.681**
	27 Discussed goals with the NGNs based on the educational program.	-.042	-.044	.168	.141	**.621**

(Cronbach’s α =.856)						
Interfactor correlation	1	1.000				
	2	.470	1.000			
	3	.465	.521	1.000		
	4	.491	.052	.056	1.000	
	5	.650	.418	.530	.350	1.000

Cumulative contribution ratio：59.572% Cronbach’s α (all)：.924 Kaiser-Meyer-Olkin (KMO) measures：.867 Bartlett's sphericity test：$p<.001$

Factor Extraction Method: Maximum Likelihood Method Rotation Method: Promax Method with Kaiser Normalization

2
