# Supplementary material for: Testing the reliability and validity of a newly graduated nurses’ teaching experience scale
Source: PLoS One. 2026 Feb 20;21(2):e0343270. doi: 10.1371/journal.pone.0343270 (PMC12923036; doi:10.1371/journal.pone.0343270)
Supplement: S3 Table — (PDF) [file pone.0343270.s003.pdf]

Table 3. Reliability analysis of the Newly Graduated Nurses Teaching Experience Scale

Factor	Items	Corrected Item Total Correlation	Cronbach's α coefficient if item is deleted	Cronbach's α (All: .924)
Factor 1	1 Seniors directly communicated with me about the NGNs' growth.	.680	.919	.923
	2 Seniors sympathized with my experience teaching NGNs.	.673	.919	
	3 Seniors asked me if I had any problems while teaching NGNs.	.565	.921	
	4 I received advice from seniors on how to instruct NGNs.	.698	.919	
	5 I talked to seniors about my problems with teaching.	.669	.919	
	6 I consulted with my seniors about specific guidance methods for NGNs.	.744	.918	
	7 I asked my seniors to provide guidance to NGNs when I was not available.	.730	.918	
	8 Nurses with less experience in teaching newly graduated nurses encouraged each other.	.641	.920	
Factor 2	9 I tried to create an atmosphere where NGNs felt comfortable talking to me about things other than work.	.255	.925	.854
	10 I tried to empathize with NGNs' stories.	.255	.925	
	11 I told NGNs that I could be a consultant for them.	.562	.921	
	12 I paid attention to the relationship between the NGNs and other staffs.	.553	.921	
	13 I ensured that the NGNs and I could openly express our thoughts to each other.	.543	.921	
	14 I felt that I was in charge of the NGNs and that I cared about the NGNs.	.573	.921	
	15 I made time for the NGNs to tell me what they were worried about in their work.	.594	.920	
Factor 3	16 I felt that my perspective on education was broadened.	.550	.921	.865
	17 I felt glad to have been involved in the training NGNs.	.470	.922	
	18 I gained confidence in education.	.351	.924	
	19 Through the NGNs' guidance, I came to recognize myself as I am now.	.565	.921	
	20 I could see positive changes in NGNs through my guidance.	.446	.923	
Factor 4	21 I was troubled because I could not understand NGNs' ideas.	.319	.925	.849
	22 I had a difficult time communicating with NGNs.	.307	.925	
	23 I was troubled because I could not observe the NGNs' positive attitude toward learning.	.401	.924	
	24 I was troubled by the teaching method due to differences from the basic education course.	.414	.923	
Factor 5	25 Goals were set/modified each time based on the NGNs' achievement status.	.700	.919	.856
	26 Progress of knowledge and skills was checked with the NGNs.	.612	.920	
	27 Discussed goals with the NGNs based on the educational program.	.588	.921	
