# Supplementary figures and images for: Testing the reliability and validity of a newly graduated nurses’ teaching experience scale
Source: PLoS One. 2026 Feb 20;21(2):e0343270. doi: 10.1371/journal.pone.0343270 (PMC12923036; doi:10.1371/journal.pone.0343270)

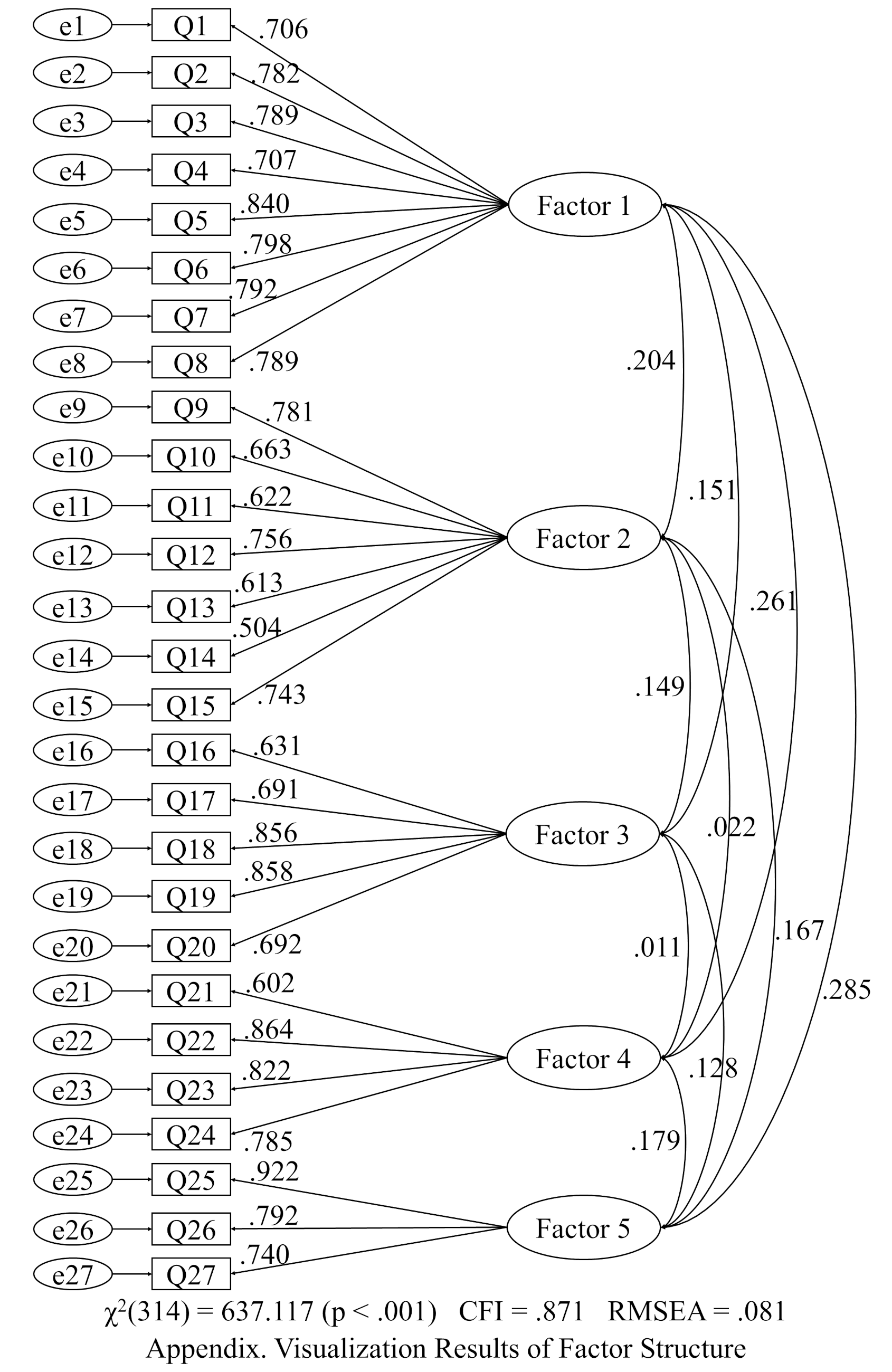

Supplement: S4 Appendix — (TIF) [file pone.0343270.s004.tif]
