# Supplementary material for: Testing the reliability and validity of a newly graduated nurses’ teaching experience scale
Source: PLoS One. 2026 Feb 20;21(2):e0343270. doi: 10.1371/journal.pone.0343270 (PMC12923036; doi:10.1371/journal.pone.0343270)
Supplement: Attachment — Submitted filename: reviewer_comments_and_the_answers.docx [file pone.0343270.s006.docx]

**Reviewer #1**: Review of the Manuscript: "Testing the Reliability and Validity of a Newly Graduated Nurses Teaching Experience Scale

Conceptual Clarity & Applicability

The focus of the study on assessing nurses' teaching experiences seems more appropriate for qualitative exploration. Identifying teaching experiences through qualitative methods would provide a more comprehensive understanding. Following this, the development of an instrument based on specific characteristics and conditions required for nurses to act as educators could have been more effective. As a result, I was unable to establish a clear connection with the instrument's practical application as presented in the current form of the paper.

→As you pointed out, this study has some limitations. To enhance its practical applicability, further research is needed with larger sample sizes and expanded recruitment of participants and facilities. This point has been added to the Limitations section.

Writing Quality

The manuscript would benefit from an improvement in its writing style. In some sections, the tone resembles a report rather than a scientific article. This can be adjusted to make the paper more formal and aligned with the expectations of scholarly writing.

→We thoroughly reviewed the entire manuscript and sought the services of a professional English-language editing company (Editage) to improve its quality.

Abstract

In the abstract, more information should be provided about the instrument, such as the number of initial items included. Additionally, mentioning the ethics committee approval in the abstract is unnecessary and should be omitted.

→We added the initial number of items to the abstract and removed the part about ethical considerations.

Content Validity

I noticed that there is no mention of content validity analysis (CVI & CVR). Including a thorough discussion of these measures would significantly strengthen the paper’s methodological rigor.

→We added the following to the Data Analysis section: “Furthermore, the research members involved in nursing education repeatedly discussed and confirmed the validity of each item.”

Software for CFA

Could you clarify which software was used for the Confirmatory Factor Analysis (CFA)? Providing this information will enhance the transparency of your methodological approach.

→We clarified this in the revised manuscript.

Sample Size and Limitations

While your sample size meets the minimum requirement, it would be beneficial to acknowledge a potential limitation. Since both Exploratory Factor Analysis (EFA) and CFA were performed on the same sample, it would be prudent to discuss this as a limitation in your study’s design.

→We now describe the limitations of the analysis due to the small sample size in the Limitations section. Additionally, we revised the section on CFA based on Reviewer 2’s comments.

**Reviewer #2**: The manuscript is valuable in its intent, but the conceptualization of the construct and the statistical analysis require revision. First of all, it is a major methodological error to perform EFA and CFA on the same sample. There is no mention of dividing the sample into two subsamples. When CFA is conducted on the same dataset used for EFA, CFA is no longer confirmatory analysis but only a circular verification. Moreover, on a sample of 157 people in the case of an instrument with 27 items, it is not advisable to perform CFA. The authors attempt to justify this by citing an author who suggests a ratio between the number of participants and the number of factors (lines 262-263). The 20:1 ratio is an outdated empirical rule applicable to EFA, not CFA. CFA is not based on the ratio of the number of participants per factor but on the complexity of the model and the degree of saturation. In CFA, the general guideline rule is 10 participants (and, according to some authors, even 25) per estimated parameter. Recent literature (e.g., Kline, 2016; Hair et al., 2022) offers much more demanding standards than Paul K (1993) proposes. It seems to me that this justification appears post hoc and seems intended to rationalize a questionable methodological decision, especially given that the authors acknowledge that the sample is small.

→Thank you for your comments. We have now included the results of the exploratory factor analysis. For reference, we checked for potential issues with the factor structure and found no major problems; therefore, we included it as an appendix. We believe the results of this exploratory factor analysis may, to some extent, reflect the current state of instructional experience. We therefore added this point to the Discussion section. However, we recognize that further investigation is necessary for generalizability and have added this to the Limitations section.

Conceptual inconsistency of the construct. Secondly, it is stated that it is about a Teaching Experience Scale for nurses with little teaching experience, but the dimensions identified do not describe the actual teaching experience but general social and emotional perceptions.

The authors state that they derived the items from previous literature but do not indicate which conceptual dimensions were taken over or modified.

→This study is not a hypothesis-testing design but rather an exploratory investigation into the factors that may emerge. Upon examining each factor using prior research, we concluded that these factors reflected elements characteristic of the less experienced nurses who were the current participants. We consider the ability to measure the nature of new nurse mentoring experiences to be a novel point. We appreciate your understanding that the results should be interpreted with this in mind.

The correlation with CNCSS (r=.222) is quite low, indicating very modest criterion validity. Although the authors show that it is significant, such a value explains less than 5% of the common variance.

→We re-examined the interpretation of this correlation coefficient and revised our discussion to account for the finding that it showed little correlation with nursing practice competence.

In a measure validation process, it was expected that other forms of validity (convergent, discriminant, etc.) would also be achieved.

→We believe there are limitations to the validity verification of these results, and we revised our Limitations section accordingly.

Only one comparable instrument is mentioned, the Self-evaluation Scale of Preceptor Role Performance for New Graduate Nurses (although there are others—e.g., the Preceptor Self-Efficacy Scale and the Nursing Clinical Teaching Effectiveness Inventory), but no systematic comparison is made between the instrument itself and the Self-evaluation Scale of Preceptor Role Performance for New Graduate Nurses.

→This study aimed to measure the experiences of nurses with limited years of experience through new graduate mentoring. The Preceptor Role Self-Assessment Scale was developed based on surveys of nurses with more years of experience than our participants or those with more mentoring experience, making it less likely to reflect the specific experiences of the nurses we focused on in this research. Furthermore, since nurses with a certain level of nursing practice ability, even if they have relatively little experience, often take on the role of precepting newcomers, this study verified the relationship using the correlation with the Clinical Nursing Competence Self-Assessment Scale rather than the Preceptor Role Self-Assessment Scale. However, owing to the low correlation coefficient, it was deemed insufficient for direct comparison and was therefore added to the Discussion section.

Line 239 - CFI value of 0.871 does not meet the minimum standards. The value of CFI ≥ 0.90

→We acknowledge that the CFI value did not reach 0.90 and mention this in the Results and Discussion section. Since the sample size may have influenced this outcome, further research with a larger sample is necessary. We added this point to the Limitations section.

Data collection procedures are also unclear. It is mentioned that administrators distributed the questionnaires, suggesting a paper-and-pencil format, but this must be clearly specified. It is important to also mention the environment in which the questionnaires were completed: at the hospital, at home, in working hours etc. The data are from 8 years ago (2017). In the context of post-pandemic changes, the generalizability of the data is low.

→The survey forms were distributed in paper format by hospital administrators to participants, which we added to the Data Collection section. Furthermore, since no specific location or circumstances were designated for participants to complete the forms, leaving it to their discretion, this was also added. As it is inferred that the nature of in-service education may have changed in some areas before and after the pandemic, this point was added to the Limitations section.

Table 2. - to be verified. includes a potential error: factor loading greater than 1.0

→The factor analysis rotation method used this time is Promax rotation. While Varimax rotation prevents factor loadings from exceeding 1.0, Promax rotation allows them to surpass this limit. Although the loadings exceeded 1.0 in this case, the excess amount was only 0.036. Furthermore, the maximum factor correlation was 0.650, which we consider acceptable.

Minor observation: Line 167 - an interesting way of phrasing the significance threshold!!!

→We revised as follows “…with the significance level set at α = .05.”

Major revision requested: increase the number of participants and create two subsamples. EFA to be done on one sample and CFA to be done on another sample.

→As you pointed out, the sample size is indeed small. However, there is a time gap between the existing data and the data that need to be re-collected. This means that the social context, subject attributes, and healthcare and education systems at that time may differ from those of the present. Therefore, if new data were collected, it would be gathered under different time periods and circumstances. Homogeneity cannot be guaranteed, and treating these as the same analytical subject could compromise the validity of the results. Consequently, we conducted the analysis using the original dataset. We would appreciate your understanding that these results were obtained within the recognized limitations of this study.
